# The impact of methodological and temporal variation on infarct size quantification in acute myocardial infarction with late enhancement CMR

**DOI:** 10.1186/1532-429X-17-S1-P149

**Published:** 2015-02-03

**Authors:** Nishat Siddiqi, Christopher J Neil, Jagpal Baljit, Jemma Hudson, Michael P Frenneaux, Dana K Dawson

**Affiliations:** Cardiology, University of Aberdeen, Aberdeen, UK; Health Sciences Research Unit, University of Aberdeen, Aberdeen, UK

## Background

Infarct size (IS) is one of the most important predictors of outcome after acute myocardial infarction (AMI) and can be detected in vivo with Late Enhancement CMR (LGE). However, the most consistent method of LGE quantification is yet to be determined.

## Methods

55 patients with reperfused, first acute ST-elevation AMI underwent LGE-CMR on a Philips Achieva 3T scanner at 1 week and 6 months post AMI. IS was expressed as a percentage of LV volume and measured at both time points using: manual planimetry, signal intensity threshold indicating LGE set at 2, 3 and 5 standard deviations (SD) above the remote myocardium and the full width at half maximum (FWHM) technique, which uses half the maximal signal within the scar as the threshold. The relationship between all measures of IS and final (6 month) LV ejection fraction (LV EF) and LV end diastolic volume (LV EDV) was evaluated using Spearman correlations.

## Results

Mean age was 61±11 years and 63% were male. All techniques showed a consistent reduction in IS between the early and six month scans (Figure) with the difference of early-to-late mean IS in decreasing order of: 10.3% for FWHM, 10% for manual, 8.5% for 2SD, 4.6% for 3SD and 1.4% for 5SD - which showed least time-dependent variation.

**Figure 1:**
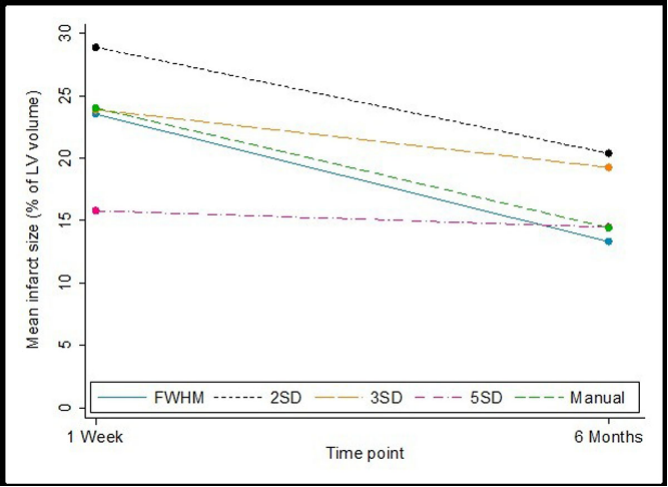
Mean IS at one week and six months using the different methods

Inter and intra observer variabilities using Intra-class Correlation (ICC) were ≥0[ddawson1] .9 for all methods (p<0.001).

As a general characteristic, all IS methods were better predictors of final LV EF than final LVEDV (Tables 1 and 2). On the early scan manual planimetry performed best as a predictor of 6 month LVEF whereas on the 6 month scan FWHM performed best as a correlate of LVEF on the same scan (Table 2).

**Table 1:** Spearmans Correlation Coefficient of IS measured at 1 week and EF and EDV at 6 months and IS and EF and EDV at 6 months (p<0.001 for all)

METHOD	EF 6 MONTHS	EDV 6 MONTHS	METHOD	EF 6MONTHS	EDV 6 MONTHS
FWHM 1 WEEK	-0.55	0.38	FWHM 6 MONTHS	-0.61	0.42
2SD 1 WEEK	-0.53	0.53	2SD 6 MONTHS	-0.52	0.41
3SD 1 WEEK	-0.52	0.53	3SD 6 MONTHS	-0.54	0.44
5SD 1 WEEK	-0.40	0.50	5SD 6 MONTHS	-0.59	0.48
MANUAL 1 WEEK	-0.61	0.48	MANUAL 6 MONTHS	-0.49	0.41

## Conclusions

FWHM, manual planimetry and 5SD had good agreement for final (6 month) IS, and 5SD showed least time-dependent variation. Manual planimetry is a better predictor of final EF when measured early whereas FWHM is a better correlate of EF when measured form late scans.

## Funding

This project was funded by a grant from the MRC(UK).

